# Detection of metastases in sentinel lymph nodes of breast cancer patients by multiple mRNA markers

**DOI:** 10.1038/sj.bjc.6601659

**Published:** 2004-03-30

**Authors:** B Weigelt, P Verduijn, A J Bosma, E J Rutgers, H L Peterse, L J van't Veer

**Affiliations:** 1 Division of Experimental Therapy, The Netherlands Cancer Institute, Plesmanlaan 121, 1066 CX Amsterdam, The Netherlands; 2 Department of Surgery, The Netherlands Cancer Institute, Plesmanlaan 121, 1066 CX Amsterdam, The Netherlands; 3 Division of Diagnostic Oncology, The Netherlands Cancer Institute, Plesmanlaan 121, 1066 CX Amsterdam, The Netherlands

**Keywords:** sentinel lymph nodes, metastases, breast cancer, quantitative real-time PCR

## Abstract

Disseminated breast tumour cells in sentinel lymph nodes (SNs) were evaluated by quantitative real-time PCR and the sensitivity of this assay was compared to the routine histological analysis. First, several candidate marker genes were tested for their specificity in axillary lymph nodes (ALN) of 50 breast cancer patients and 43 women without breast cancer. The marker gene panel selected, designed to detect the mRNA of CK19, p1B, EGP2 and SBEM, was subsequently applied to detect metastases in 70 SNs that were free of metastases as determined by standard histological evaluation. Remarkably, seven negative SNs showed increased marker gene expression, suggesting the presence of (micro) metastases. Four of these seven SNs positive by real-time PCR proved to contain tumour deposits after careful review of the slides or further sectioning of the paraffin-embedded material. In three PCR positive SNs, however, no tumour cells were found by haematoxylin and eosin staining (H&E) and immunohistologically analysis. The quantitative real-time PCR assay with multiple mRNA markers for the detection of disseminated breast cancer cells in SNs thus resulted in an upstaging of SNs containing metastastic disease of 10% compared to the routine histological analysis. The application of this technique may be of clinical relevance, as it is suggested that micrometastatic disease in SNs are associated with further nodal non-SN metastases in breast cancer.

In breast cancer to date, the axillary lymph node (ALN) status remains the most valuable individual prognostic factor for disease course and recurrence (McGuire, 1987; Foster, 1996). Involvement of lymph nodes and the number of lymph nodes harbouring metastases at primary diagnosis have an inverse relationship with the disease prognosis, meaning that patients with lymph nodes free of metastases have a better outcome (Fisher *et al*, 1983; Carter *et al*, 1989; Hellman, 1994; Quiet *et al*, 1995; Saimura *et al*, 1999). However, 20–30% of node-negative patients will develop a relapse in 5–10 years after diagnosis (Kamby *et al*, 1991; Hellman, 1994). In addition, it is known that 20–30% of node-positive patients are long-term survivors (Rosen *et al*, 1989; Joensuu *et al*, 1998). Based on these obvious shortcomings of the lymph node status, new procedures and markers are continuously investigated with regard to their prognostic value. Recently, it was shown that primary tumours themselves already contain a gene expression profile that is strongly predictive of metastasis and poor survival (van de Vijver *et al*, 2002; van't Veer *et al*, 2002), thereby challenging treatment choice based on routine prognostic markers in the future. However, until gene-expression profiling will increasingly be used for clinical decision-making, the nodal status remains a main prognostic factor.

Until recently, the standard treatment for patients with operable breast cancer included the complete dissection of 10–30 ALN. More than half of these patients were found to have metastases-free lymph nodes and thus had been subjected to unnecessary surgical risks and complications (Giuliano *et al*, 1997). A less invasive method for the assessment of lymph node status is the sentinel lymph node biopsy (SLNB). Thereby, the lymphatic route of tumour cells to the lymph node(s) that primarily drains the tumour and most likely harbours metastatic disease are mapped (Giuliano *et al*, 1994). The sentinel lymph node (SN) is highly predictive of the histo-pathology of the remaining lymphatic basin and can accurately predict axillary nodal status in at least 98% of cases (Giuliano *et al*, 1995, 1997). Furthermore, the SN biopsy allows a more extensive and focused search for metastases in one or two nodes, in contrast to the present limited analysis of the multiple lymph nodes from a complete dissection. Multiple step sectioning and immunohistochemistry staining of the SN increases the accuracy of axillary staging in breast cancer patients compared with axillary lymph node dissection (ALND) plus routine histo-pathologic examination of lymph nodes (Giuliano *et al*, 1995). In this way additional micrometastases are identified, which may relate to prognosis (Bourez and Rutgers, 2001; Tjan-Heijnen *et al*, 2001). The SN procedure therewith provides an accurate and feasible alternative for complete ALND as a staging tool in breast cancer and can prevent lymph node negative women from unnecessary surgery (Bourez *et al*, 2002).

In the study presented, a sensitive real-time PCR approach is used for the detection of metastases in SNs. We have examined several candidate marker genes, with particular emphasis on sensitivity and specificity of these genes in ALN of patients with and without metastases. Finally, a marker panel of four genes comprising cytokeratin 19 (CK19), trefoil factor-3 (p1B), epithelial glycoprotein-2 (EGP-2) and small breast epithelial mucin (SBEM) was used to compare the quantitative real-time PCR detection method to the routine analysis of SNs, including multiple step sectioning and immunohistochemical staining.

## MATERIAL AND METHODS

### Axillary and SN specimens from breast cancer patients

Axillary (*n*=50) and SNs (*n*=89) of breast cancer patients were selected from the fresh-frozen tissue bank of the Netherlands Cancer Institute/Antoni van Leeuwenhoek hospital (NKI/AvL). As negative controls 43 ALNs were obtained from patients without breast cancer undergoing a preventive breast ablation or a lymph node biopsy for analysis of lymphoma. The latter group was classified to have benign enlarged lymph nodes. The SN procedure was introduced in 1996 at the NKI/AvL (Rutgers *et al*, 1998) and since then performed on more than 700 patients. The SN was removed after localisation of the node by combination of radioactivity measurement by a gamma detection probe (Neoprobe 100/1500, Neoprobe Corporation, Dublin, UK) exploiting the remaining activity after preoperative lymphoscintigraphy, and visually by peroperative injection of Patent Blue V (Blue Patente V; Laboratoire Guerbet, Aulnay-sous-Bois, France) (Doting *et al*, 2000).

The ALNs obtained at preventive breast ablation and the lymph nodes retrieved from analysis of lymphoma were bisected, snap frozen in liquid nitrogen and stored at −80°C. SNs were subjected to frozen section evaluation. Sentinel lymph nodes up to 0.5 cm were completely embedded in Tissue Tek (Sakura Finetek, Zoeterwoude, The Netherlands), larger SNs were bisected or lamellated in slices of 0.2 cm and separately sectioned. Frozen section cutting was aimed at obtaining a complete cross section at a single level and preventing tissue loss. Before the frozen section procedure, the microtome was carefully cleaned to avoid contamination; all lost tissue was collected and stored at −80°C. The amount of lost tissue varied, and was estimated at least 20 sections of 10 *μ*m. Remaining SN tissue was examined after formalin fixation and paraffin embedding by haematoxylin and eosin (H&E) staining and immunohistochemistry at three levels (150 *μ*m distance). CAM 5.2 (Becton Dickinson, San Jose, USA) was used as antibody.

### RNA isolation and quantitative real-time PCR

RNA was isolated from 30 tissue sections of 30 *μ*m thickness made from each axillary node, and from the lost frozen tissue of the SNs, using RNAzol B (Campro Scientific, Amersfoort, The Netherlands). In total, 1 *μ*g total RNA was used for cDNA synthesis (20 *μ*l), as described previously (Lambrechts *et al*, 1999).

Based on the published genomic sequences of CK19, p1B, EGP-2, PS2, mammaglobin and SBEM, the sequences of the real-time quantitative PCR primers (Sigma Genosys, Cambridge, UK) and of the 5′-fluorescently FAM labelled probes (Applied Biosystems, Nieuwerkerk a/d IJssel, The Netherlands) were selected using the Perkin-Elmer Primer Express® software (PE, Foster City, USA) (Table 1

**Table 1:** Primer and probe sequences of each breast cancer marker gene for real-time PCR amplification. All sequences are written 5′ → 3′

**Marker gene**	**Genbank accession no.**	**Primers**	**Probe (5′FAM-3′TAMRA)**
p1B	(L15203)	Sense: CTGAGGAGTACGTGGGCCTG	CTGCAAACCAGTGTGCCGTGCC
		Antisense: AGTCCACCCTGTCCTTGGC	
PS2	(X00474)	Sense: GAGGCCCAGACAGAGACGTG	CTGCTGTTTCGACGACACCGTTCG
		Antisense: CCCTGCAGAAGTGTCTAAAATTCA	
CK19	(NM002276)	Sense: CTACAGCCACTACTACACGAC	CACCATTGAGAACTCCAGGATTGTCCTGC
		Antisense: CAGAGCCTGTTCCGTCTCAAA	
EGP2	(M32306)	Sense: CAGTTGGTGCACAAAATACTGTCA	TTGCTCAAAGCTGGCTGCCAAATGTT
		Antisense: CCATTCATTTCTGCCTTCATCA	
SBEM	(AF414087)	Sense: CTCTTGGGGAGTTTTCCATCTTTCTG	CCCAGAATCCGACAACAGCTGCTCC
		Antisense: CTTCATCATCAGCAGGACCAGTAG	
MaGl	(AF015224)	Sense: TTCTTAACCAAACGGATGAAACTCT	TGCTGTCATATATTAATTGCATAAACACCTCAACATTG
		Antisense: GGTCTTGCAGAAAGTTAAAATAAATCAC	

). All primers were designed to be intron-spanning to preclude amplification of genomic DNA. To normalise relative levels of expression, commercially available primers and probes for the housekeeping genes, glyceraldehyde-3-phosphate dehydrogenase (GAPDH) and *β*-actin were used (Applied Biosystems).

Serially diluted cDNA synthesised from RNA isolated and pooled from 6 × 10^6^ cells of five breast cancer cell lines (MCF7, CAMA, T47D, MPL13, SkBr3 (American Tissue Culture Collection, Rockville, USA)), respectively, was used to generate standard curves for control and marker gene expression. For all cDNA dilutions, the fluorescence was detected from 0 to 50 PCR cycles for the control and marker gene in singleplex reactions and resulted in the *C*_T_-value for each cDNA dilution and each target (*C*_T_-value (threshold cycle): the PCR cycle at which a significant increase in fluorescence is detected, due to the exponential accumulation of PCR products, represented in arbitrary units (TaqMan Universal PCR Master Mix Protocol, Applied Biosystems) (Bieche *et al*, 1999). The quantities found for the GAPDH control and marker gene were used to calculate the relative quantity of control and marker gene expression in ALN and SNs. The second control gene, *β*-actin, was only used for the confirmation of GAPDH expression. Each experiment was performed in triplicate. The quality control of the PCR reactions was assessed by standardised PCR conditions, including in each experiment a genomic DNA control and a negative nontemplate control.

### Statistics

The QDA score function, as defined earlier (Bosma *et al*, 2002), was calculated from the expression levels of the six marker genes CK19, p1B, EGP-2, SBEM, PS2 and mammaglobin tested, in different combinations. QDA is a statistical technique to find the combination of quadratic and linear functions of variables (e.g. marker genes), which leads to an optimal separation between groups (e.g. tumour cell positive and negative lymph nodes). It is a generalisation of the more familiar Fisher's Linear Discrimination Analysis (LDA), which allows only linear functions. QDA performs better than LDA if the groups differ not only with respect to the means of the variables but also with respect to standard deviations and/or correlations. A positive discriminant score of the four marker genes selected, CK19, p1B, EGP-2 and SBEM, indicates the presence of breast tumour cells, a negative discriminant score indicates the absence of tumour cells. The discriminant threshold score separating these two groups is zero.

### Sensitivity

A cDNA pool of three tumour cell positive ALNs was diluted 1 : 10, 1 : 100, 1 : 1000, 1 : 10 000 and 1 : 100 000 in a cDNA pool of three control ALNs. For all cDNA dilutions the fluorescence was detected from 0 to 50 PCR cycles, for each individual marker gene in triplicates. The *C*_T_-values obtained were compared to serially diluted cDNA synthesised from RNA isolated from 5000 MCF7 cells. The dilution of MCF7 RNA of 1 : 10, 1 : 100, 1 : 1000, 1 : 10 000 and 1 : 100 000 corresponds to the total RNA amount obtained from 500, 50, 5, 0.5 and 0.05 MCF7 cells, respectively. All standard curves for these experiments were generated using the cell line mix as described for the marker and control gene real-time PCR reactions.

## RESULTS

### Marker gene selection for the detection of metastatic breast tumour cells in ALN

Several potential marker genes were tested to determine genes that are highly expressed in 50 ALNs of breast cancer patients with pathological verified tumour involvement, but that are at the same time expressed at low levels in 43 ALNs of women without evidence of breast cancer. Gene expression was quantitated for the four breast cancer marker genes CK19, PS2, EGP-2 and p1B, which we used earlier for the detection of circulating tumour cells in peripheral blood of breast cancer patients (Bosma *et al*, 2002). Furthermore, the genes mammaglobin, epidermal growth factor receptor (EGFR) and SBEM were tested, described in the literature as breast cancer marker genes (De Luca *et al*, 2000; Mitas *et al*, 2001; Miksicek *et al*, 2002; Zehentner *et al*, 2002). For each gene, *C*_T_-values for negative control lymph nodes and metastatic breast cancer lymph nodes were obtained from triplicate reactions. For all ALN samples, the expression level for each of the marker genes relative to the breast cancer cell line mix standard curve was calculated and corrected for the input of cDNA based on the GAPDH control (see Materials and Methods) (Table 2

**Table 2:** Expression levels (quantities) for each housekeeping gene relative to the standard curve and relative quantities for each marker gene relative to the standard curve and corrected for the input of cDNA based on the GAPDH control in arbitrary units (au)

**Housekeeping gene**	**Tissue**	**Median quantity**	**Range**
GAPDH	ALNneg	0.30000	0.01800–1.85000
	ALNpos	0.15500	0.00002–1.90000
*β*-Actin	ALNneg	0.76250	0.09800–2.75000
	ALNpos	0.28000	0.00001–1.35000
			
**Marker gene**	**Tissue**	**Median relative quantity**	**Range**
CK19	ALNneg	0.00001	0.000003–0.000103
	ALNpos	0.04931	0.00022–8.23529
p1B	ALNneg	0.01778	0.00009–0.23583
	ALNpos	0.13400	0.00017–55.5556
EGP2	ALNneg	0.00035	0.00009–0.00270
	ALNpos	0.07300	0.00112–0.69109
SBEM	ALNneg	0.00047	0.00011–0.02112
	ALNpos	0.07800	0.00073–84.7619
PS2	ALNneg	0.00001	0.00000–0.00026
	ALNpos	0.01319	0.00000–10.0457
Mammaglobin	ALNneg	0.000001	0.00000–0.00003
	ALNpos	0.00014	0.00001–1.16994
EGFR	ALNneg	3.06956	0.80620–10.1852
	ALNpos	1.45317	0.05461–58.2897

ALNneg=tumour cell free control axillary lymph nodes; ALNpos=axillary lymph nodes harbouring breast tumour cells.

).

**Figure 1:**
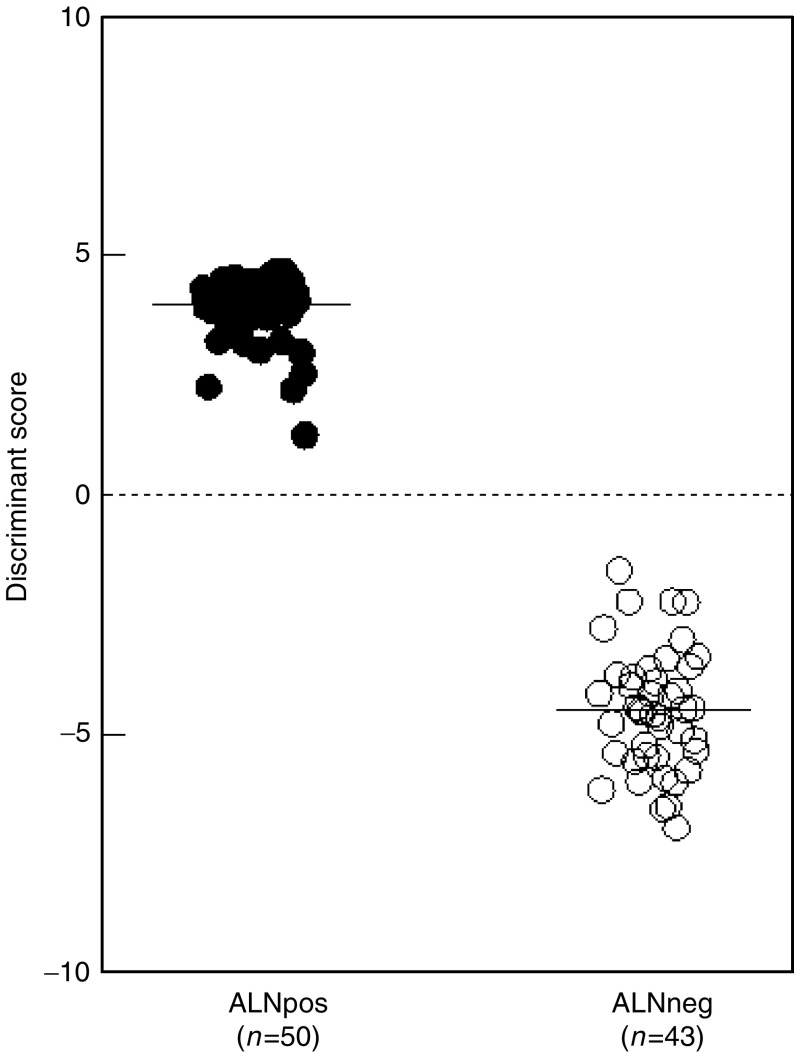
Discriminant score of expression of the four marker genes CK19, p1B, EGP2 and SBEM in ALN of patients with and without breast cancer. The median expression levels for the markergene panel within a group are indicated by a horizontal line. Closed circles represent breast cancer patients, open circles women without breast cancer. The discriminant score separating the two groups is indicated by a dashed line.

We observed that the median expression levels of EGFR were higher in the control ALNs than in the tumour cell positive lymph nodes (Table 2). Thus, this gene was not useful for the detection of metastatic breast cancer in lymph nodes, whereas the median expression levels for CK19, p1B, EGP-2, SBEM, PS2 and mammaglobin were significantly higher in the ALNs containing metastatic breast cancer (Table 2), determined by the Mann–Whitney test (data not shown). To optimally use the expression levels of these latter marker genes to separate lymph nodes with and without tumour cell involvement, the quadratic discriminant analysis (QDA) was employed (Bosma *et al*, 2002) (see Material and Methods). A combination of four marker genes was shown to have the highest specificity in separating tumour cell negative and positive lymph nodes (data not shown). In the following step, we tested different combinations of sets of four genes to determine the marker gene panel that gave the largest discriminant score between negative and tumour cell positive lymph nodes. The marker set including CK19, p1B, EGP2 and SBEM gave the largest separation between breast tumour cell negative and positive ALNs with zero misclassified normal control lymph nodes (Figure 1

). The median for the positive and negative lymph nodes in the discriminant score is 4.05 (range: 1.25–467) and −4.51 (range: −6.97 to −2.79), respectively (*P*-value <0.0001). The threshold score discriminating the two groups is zero. A cross validation also showed a 100% separation between the tumour cell positive and negative ALNs (data not shown). These results indicate that the real-time PCR analysis using a marker panel of genes is specific and at least as sensitive as the standard histological analysis.

### Sensitivity

**Figure 2:**
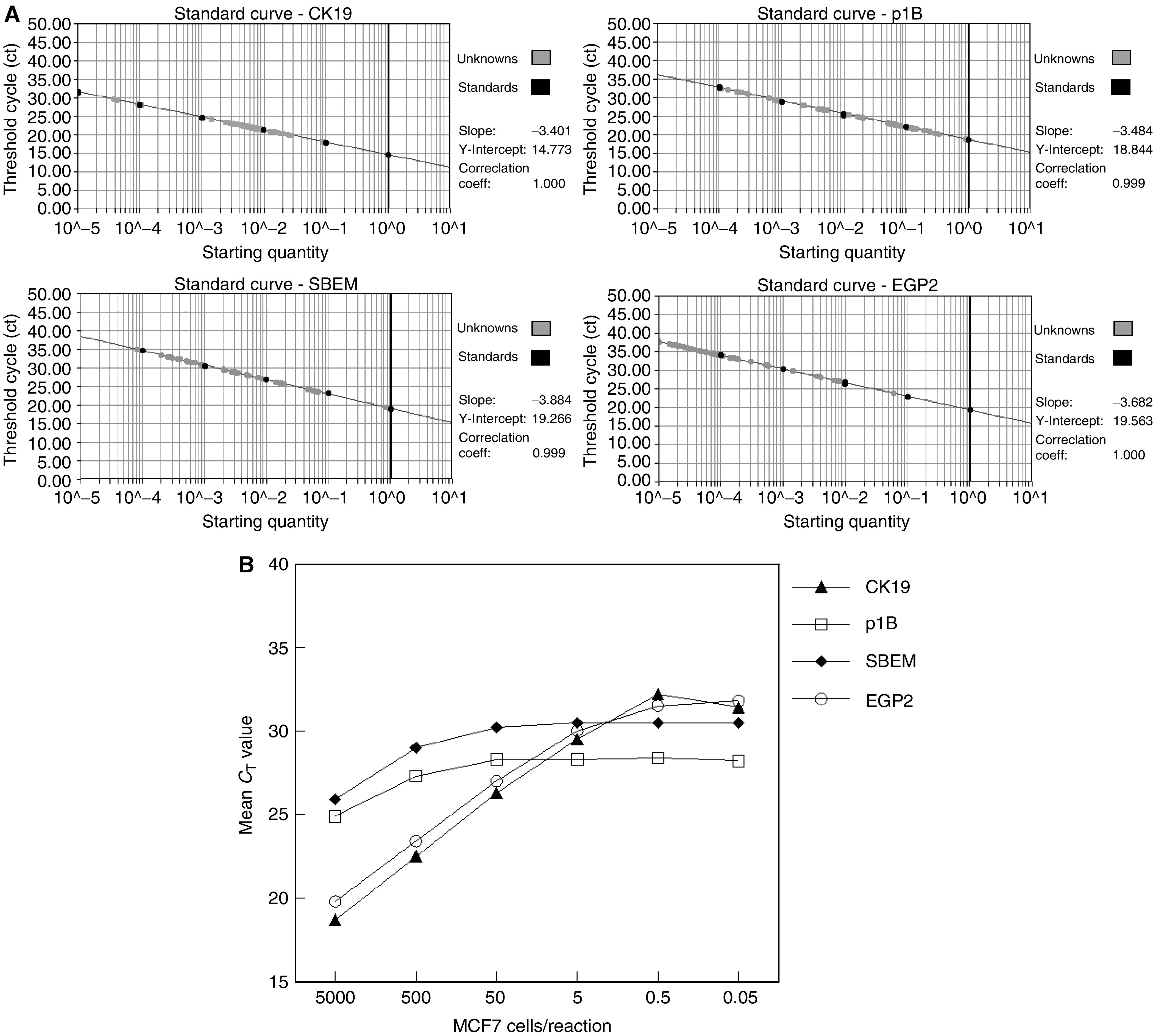
(**A**) Standard curves of CK19, p1B, EGP2 and SBEM, used for determining the sensitivity of the marker genes (black circles). Grey circles represent lymph node samples tested. The threshold cycle (*C*_T_-value) is plotted against the starting quantity of a pool of five breast cancer cells lines. (**B**) Sensitivity of the markerpanel CK19, p1B, EGP2 and SBEM real-time PCR defined by analysis of serial dilution of a cDNA pool of three tumour cell positive ALN in a cDNA pool of three control lymph nodes. Subsequently, *C*_T_-values obtained were compared to serially diluted MCF7 cDNA, for each marker gene individually. The detection limit of p1B with regard to the background expression of control lymph nodes corresponds to the amount of 50 MCF7 cells, for SBEM to five MCF7 cells and for CK19 and EGP2 to 0.5 MCF7 cells.

To define the value of the quantitative real-time PCR analysis for the detection of metastatic breast cancer cells, we determined the sensitivity of the marker panel. Working with a solid tissue such as lymph nodes makes it difficult to dilute breast tumour cells directly in the tissue like it is usually done for sensitivity assays in peripheral blood (Kufer *et al*, 2002). Instead, we pooled cDNA of three tumour cell positive ALNs and serially diluted this cDNA corresponding to five log steps in a cDNA pool of three control ALNs. A real-time PCR was performed for all cDNA dilutions, for each marker gene individually, and the detection limit of the assay was defined. To get an indication of how many breast tumour cells can be detected using the quantitative PCR approach, we compared the *C*_T_-values obtained to those of serially diluted cDNA synthesise from RNA isolated from 5000 MCF7 cells. The dilution of 10^−1^ up to 10^−5^ corresponds to 500 to 0.05 MCF7 cells, respectively. The standard curves for all experiments were generated from a pool of five breast cancer cell lines, which expressed all four marker genes tested at similar high levels (Figure 2A

). The limit of detection of the cDNA pool of tumour cell positive lymph nodes diluted in a pool of negative lymph nodes is shown in Figure 2B for every of the four marker genes individually. The *C*_T_-value of p1B reaches a plateau when less than 50 MCF7 cells are present, indicating that a sensitivity of 50 breast tumour cells can be reliably detected by this marker. The detection limit for SBEM corresponds to the presence of five MCF7 cells and for CK19 and EGP2 to as low as 0.5 MCF7 cells (Figure 2B).

### Marker gene expression in SNs

To determine whether real-time PCR analysis was also capable of detecting metastases in SNs, we analysed 70 SNs without evidence of metastatic breast tumour cells following standard histological analysis. The median discriminant score for the tumour cell negative SNs was, as for the control ALNs, smaller than zero, the threshold score that separates the tumour cell negative from the tumour cell positive ALN group. Additionally, 19 histologically proven positive SNs were analysed using the marker gene panel to stage the accuracy of the real-time approach. The median discriminant score is lower compared to the tumour cell positive ALNs. However, the set of marker genes is still over expressed in the positive SNs and the discriminant scores of all 19 SN are above the threshold of zero, indicating the presence of metastatic breast tumour cells. The marker panel containing CK19, EGP-2, p1B and SBEM can therefore also be applied for the evaluation of SNs.

**Figure 3:**
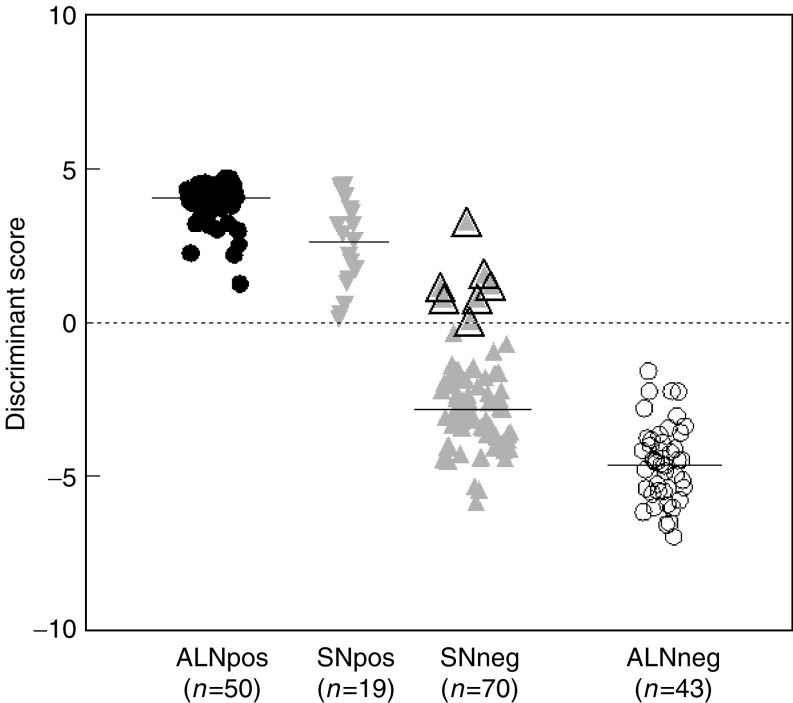
Discriminant score of expression of the four marker genes CK19, p1B, EGP2 and SBEM in ALN of patients with and without breast cancer, and in histologically negative and positive SN of breast cancer patients. The median expression levels for the markergene panel within a group are indicated by a horizontal line. Seven of the 70 histological negative SN show a positive discriminant score, indicated by a surrounding black triangle. The discriminant score separating the tumour cell positive and negative lymph nodes is indicated by a dashed line.

Remarkably, the evaluation of the 70 histologically negative SNs on an individual basis showed for seven SNs a positive discriminant score of the four mRNA marker genes, indicating the presence of metastatic tumour cells (Figure 3

). Subsequently, the frozen tissue and paraffin sections of these seven SNs, tumour cell positive by real-time PCR, were reviewed. The slides of two cases revealed tumour deposits at review, which were missed at the first evaluation. In one of these SNs, tumour was present in the frozen section slide, in the other one in the H&E stained slide of the first level. In both cases the deposits were cell clusters with a diameter smaller than 0.2 cm (micrometastases). No evidence of metastases was found in the slides of the standard evaluation of the SNs of the other five cases. The remaining paraffin-embedded material of these nodes was completely step sectioned at intervals of 50 *μ*m, stained by H&E and immunohistochemistry and revealed micrometastases in one case, whereas the other four nodes remained negative. In one of these latter patients with a tumour cell negative SN, however, a non-SN removed during the SN procedure showed to harbour breast tumour cells verified by standard histological analysis. Therefore, the possibility that the histologically negative SN also harbours metastatic cells might be high, as the positive discriminant score of the marker panel obtained by real-time PCR indicates.

## DISCUSSION

In this study, we have established a highly sensitive and quantitative multimarker real-time PCR approach to detect disseminated breast cancer cells in ALNs and SNs. Based on experiences it is unlikely that false positive results can be avoided if only a single marker gene is used in a RT–PCR based test system (Lambrechts *et al*, 1999). Using more than a single marker gene, as applied in our experiments, is a potential way to overcome the problem of illegitimate expression (Chelly *et al*, 1989), assuming that there is a little chance of encountering significant illegitimate mRNA of more than one gene at a time.

The marker genes tested, CK19, p1B, EGP-2, SBEM, PS2 and mammaglobin, share the characteristics that they are all expressed at high levels in ALNs harbouring metastatic breast tumour cells, but only at very low levels in lymph node tissue itself (Table 2). However, EGFR, a mRNA marker gene described for circulating tumour cell detection in peripheral blood of breast cancer patients (De Luca *et al*, 2000), appeared not to be applicable as a marker gene in lymph nodes due to its high background expression in control lymph nodes. Applying a discriminant score (QDA) showed that the marker set including CK19, p1B, EGP2 and SBEM gave the most significant separation between tumour cell negative and positive ALNs with zero misclassified control lymph nodes (*P*<0.0001).

The expression level for each of the marker genes was calculated relative to the standard curve and corrected for the input of cDNA based on the GAPDH control. The standard curves were generated from a mix of five breast cancer cell lines to assure that every marker gene tested will be expressed at high levels, therewith creating reproducible and reliable experiments (Figure 2A). A single breast cancer cell line as standard shows variation in gene expression levels of individual marker genes, a noticeable feature when determining the sensitivity of our marker panel used. The expression levels of SBEM and p1B in MCF7 cells are distinctly lower than those of CK19 and EGP2. This might be the reason for the nonlinear kinetics of the PCR reaction in the sensitivity experiment (Figure 2B), compared to the linear kinetics in the real-time PCR assays (Figure 2A), and the establishment of the amplification plateau below 30 cycles observed for SBEM and p1B.

When applying the set of marker genes to detect (micro) metastases in SNs, seven of the 70 histologically breast tumour cell free SNs showed a positive discriminant score, predicting the presence of metastatic disease. Four of these seven SNs could be histologically confirmed by more intensive review of slides or further sectioning of the paraffin-embedded material, what shows that standard evaluation is false-negative in 4%, whereas in three SNs no tumour cells were found by H&E staining and immunohistochemical analysis.

Using a real-time PCR approach we achieve an upstaging of SNs containing breast cancer metastases of 10% compared to the standard histological analysis. Our findings are in contrast to the recent results published by Schroder *et al* (2003) who found that in SNs immunostaining appears to be more sensitive/specific than quantitative PCR for breast tumour cell detection. The apparent discrepancy between our results and that of Schroder *et al* are likely due to the increased sensitivity achieved by the use of a multimarker panel in combination with the QDA score.

The follow-up times of the seven patients, whose histologically negative SNs showed a discriminant score predicting the presence of tumour cells using the real-time PCR approach, are too short to give an indication whether the upstaging of SNs reached by quantitative PCR has a prognostic value. Furthermore, it remains unknown whether micrometastatic disease in pathology-negative SNs have clinical significance. Ongoing clinical trials that will address this important issue (Grube and Giuliano, 2001; Ross, 2001). However, recently it was suggested that approximately 18% of the SNs harbouring micrometastases might be associated with further nodal non-SN metastases (Cserni *et al*, 2003). Our results provide information that could lead to a better management of breast cancer patients by reducing the rate of false-negative SNs using a quantitative real-time PCR approach with multiple mRNA markers instead of standard histological analysis for the detection of metastases in SNs.
